# RGK regulation of voltage-gated calcium channels

**DOI:** 10.1007/s11427-014-4788-x

**Published:** 2015-01-10

**Authors:** BURAEI Zafir, LUMEN Ellie, KAUR Sukhjinder, YANG Jian

**Affiliations:** 1 Department of Biology, Pace University, New York, NY 10038, USA; 2 Ion Channel Research and Drug Development Center, and Key Laboratory of Animal Models and Human Disease Mechanisms of the Chinese Academy of Sciences and Yunnan Province, Kunming Institute of Zoology, Chinese Academy of Sciences, Kunming 650223, China; 3 Department of Biological Sciences, Columbia University, New York, NY 10027, USA

**Keywords:** calcium, channel modulation, trafficking, cardiac physiology, neurobiology, beta subunit

## Abstract

Voltage-gated calcium channels (VGCCs) play critical roles in cardiac and skeletal muscle contractions, hormone and neurotransmitter release, as well as slower processes such as cell proliferation, differentiation, migration and death. Mutations in VGCCs lead to numerous cardiac, muscle and neurological disease, and their physiological function is tightly regulated by kinases, phosphatases, G-proteins, calmodulin and many other proteins. Fifteen years ago, RGK proteins were discovered as the most potent endogenous regulators of VGCCs. They are a family of monomeric GTPases (Rad, Rem, Rem2, and Gem/Kir), in the superfamily of Ras GTPases, and they have two known functions: regulation of cytoskeletal dynamics including dendritic arborization and inhibition of VGCCs. Here we review the mechanisms and molecular determinants of RGK-mediated VGCC inhibition, the physiological impact of this inhibition, and recent evidence linking the two known RGK functions.

## 1 Introduction

### 1.1 Voltage-gated calcium channels

Ca^2+^ ions play a critical role in biological processes ranging from neurotransmitter and hormone release to muscle contraction, cell division, differentiation, migration and death. In nerve and muscle cells, the principal entryways for Ca^2+^ are voltage-gated calcium channels (VGCCs). These are large multisubunit membrane proteins, whose mutations have been implicated in autism, epilepsy, migraine, cardiovascular and skeletal muscle disease, blindness, deafness, pain and other conditions.

**Figure 1:**
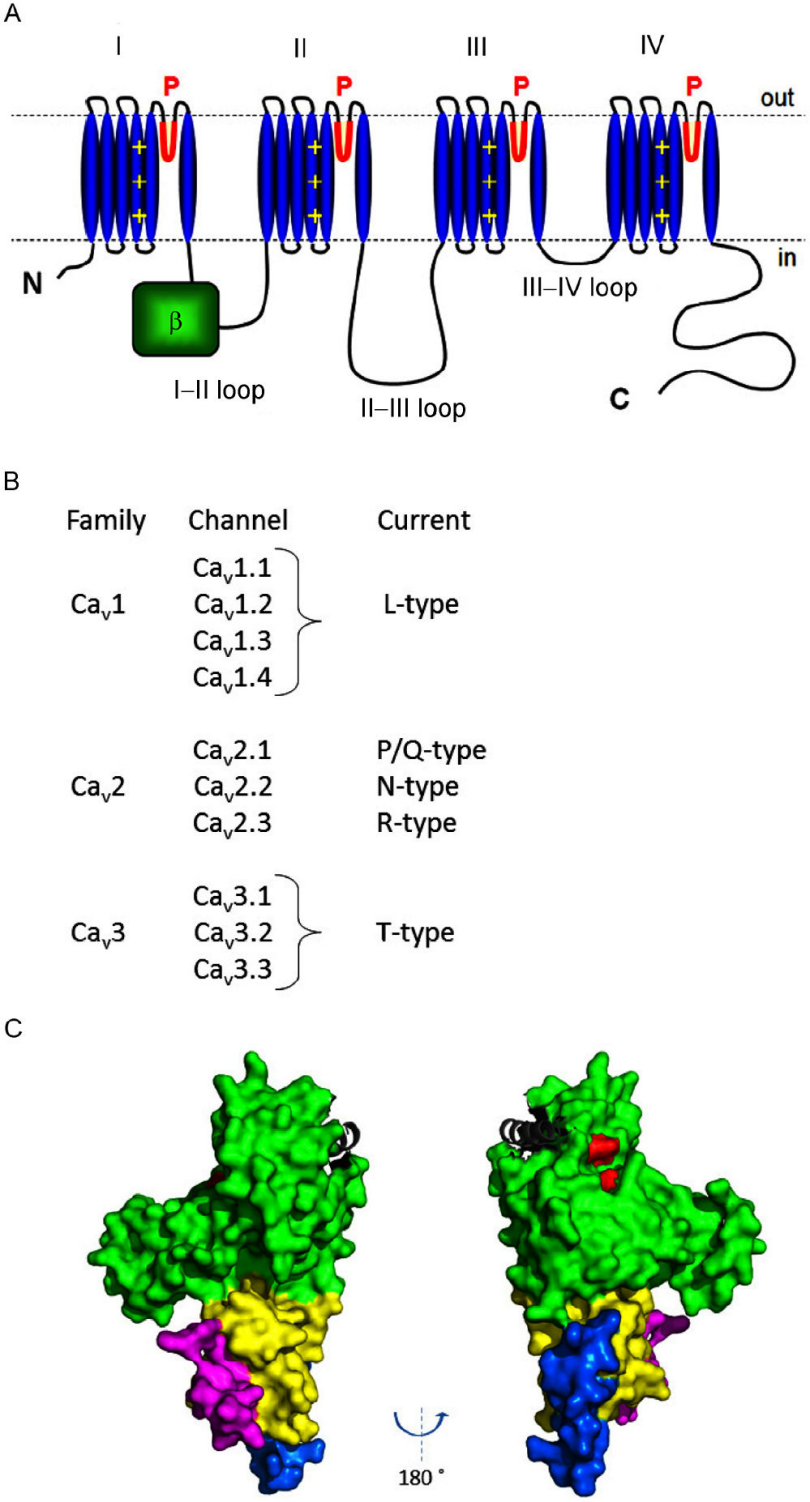
Transmembrane topology of Ca_v_α_1_, family classification of VGCCs and the crystal structure of Ca_v_β. A, The four homologous repeats of Ca_v_α_1_ are indicated by the roman numerals I–IV. Blue segments indicate transmembrane segments S1–S6, with S4 serving as the voltage sensor. The β subunit binds to the I–II loop but may also interact with other regions of the channel [3,4]. B, Classification of VGCCs. C, The crystal structure of Ca_v_β3 in complex with the AID (gray helix; PDB: 1VYT). The GK domain is in green, the SH3 domain in yellow, the HOOK region in magenta and the N-terminus in blue. In red are three aspartic acid residues (D 194, 270 and 272) thought to interact with RGK proteins.

The principal component of VGCCs is a large (~2000–2500 amino acids, 190–250 kD) pore-forming α_1_ subunit or Ca_v_α_1_. Ca_v_α_1_ has intracellular N- and C-termini and four homologous repeats (I–IV), each with six transmembrane segments (S1–S6) and a pore-forming loop. Each S4 segment contains positively charged amino acids and forms the channel’s voltage sensor, whose movement upon depolarization leads to channel opening (Figure 1). The voltage sensors’ movements elicit minuscule “gating currents” that can be measured independently from the larger Ca^2+^ currents flowing through the channel’s pore. The “gating current” concept is noted here because RGK proteins can restrict the movement of the voltage sensors in some instances [1,2]. The four homologous Ca_v_α_1_ repeats are connected by three intracellular connecting loops: the I–II loop, II–III loop and III–IV loop. The I–II loop contains the AID (α-interacting domain), which binds to the β subunit of VGCCs (Ca_v_β). As we discuss later, Ca_v_β is critical for VGCC function and inhibition by RGKs.

In mammals, distinct Ca_v_α_1_ subunits are encoded by 10 different genes with over 70 splice variants. Ca_v_α_1_ determines and defines the unique biophysical and pharmacological properties of VGCCs (Figure 1). Based on these properties, as well as sequence homology, VGCCs fall into three subfamilies: Ca_v_1, Ca_v_2 and Ca_v_3, with the subtypes shown in Figure 1. The Ca_v_1 and Ca_v_2 families are more closely related to each other than to Ca_v_3 channels. The latter are low voltage-activated (LVA) and do not have an AID in their I-II loop and do not require any auxiliary subunits for proper expression or function [3,4]. In contrast, Ca_v_1 and Ca_v_2 channels (L-, N-, P/Q- and R-type channels) generally require higher voltages for activation (HVA channels) and require auxiliary subunits for proper function. In particular, Ca_v_β plays a crucial role in trafficking channels to the plasma membrane and fine- tuning channel gating properties [3,4]. Not surprisingly, mutations and dysregulation of Ca_v_β have been implicated in long QT syndrome, Alzheimer’s disease, ataxia, cardiac hypertrophy, seizures, dyskinesia, renal cysts and other disorders [3,4].

There are four different Ca_v_βs, Ca_v_β_1_–Ca_v_β_4_, encoded by four genes that give rise to over 20 splice variants. Ca_v_βs are members of the MAGUK (membrane-associated guanylate kinases) family of proteins and have conserved GK and SH3 domains (Figure 1C), which serve as protein-protein interaction modules. In addition, they have a variable HOOK region connecting the GK and SH3 domains, as well as variable N- and C-termini that functionally distinguish different Ca_v_βs.

The conserved Ca_v_β GK domain harbors the α_1_-binding pocket (ABP), which binds to the AID and anchors Ca_v_β to the channel complex. At a separate site, the GK domain also binds and anchors RGK proteins [5] (Figure 1C). Thus, some mutations can abolish Ca_v_β-RGK binding while maintaining Ca_v_α_1_-Ca_v_β binding; vice versa, other mutations can abolish Ca_v_α_1_-Ca_v_β binding while maintaining Ca_v_β-RGK binding. These and other combinations of mutations have been exploited to dissect the role of Ca_v_β in RGK inhibition and uncover direct binding between Ca_v_α_1_ and RGKs [1,2,5,6], as we discuss below.

### 1.2 RGK proteins

All monomeric G-proteins, including RGKs, belong to the Ras superfamily of GTPases. They all have a G-domain composed of five G regions (G1–G5) involved in guanine nucleotide binding, and two regions that switch their conformation upon GTP/GDP exchange: switch I and switch II. Ras GTPases are further divided into five families, each with distinct functions: Rab, Ran, Ras, Rho, and Arf/Sar1, which are involved, respectively, in vesicular transport, nucleoplasmic transport, gene expression, cytoskeleton rear-rangements and vesicle budding [7]. In the early 1990s, the latest family of small GTPases was discovered [8]. These are the RGK GTPases Rad, Rem (also known as Rem1 or Ges), Rem2 and Gem/Kir. Rad was discovered as a Ras-like protein associated with type II diabetes, Gem as a GTP-binding mitogen-induced T-cell protein, and Rem and Rem2 were later identified based on similarity to Rad and Gem. In comparison to canonical Ras GTPases, RGK GTPases have a low or absent GTPase activity, probably due to the non-conserved amino acid substitutions in the Switch I and G3 regions [9,10]. There are, however, indications that in the presence of nm23—the only known RGK GTPase activating protein (GAP)—Rad and Gem have an enhanced GTPase activity [11]. Thus, the unique mechanism of GTP hydrolyses remains to be determined for RGK proteins. While most Ras GTPases undergo lipid modifications that help anchor them to the membrane, RGK proteins have extended C-termini that take on this role, as well as serve as hubs, together with the N-termini, for interactions with other proteins, such as calmodulin [7,12,13].

RGK proteins have two known functions: shaping cytoskeletal dynamics and inhibiting HVA Ca^2+^ channels [14]. The two RGK functions can be regulated separately, so that RGK modification of cytoskeletal reorganization, but not inhibition of HVA Ca^2+^ channels, is attenuated by dephosphorylation of certain RGK residues [15,16]. Recent findings, however, have revealed Ca_v_α_1_ as a point of convergence for the two functions. Namely, RGK binding to VGCCs seems to be critical for regulating cytoskeletal dynamics and dendritic arborization of nerve cells [17].

## 2 RGKs inhibit Ca_v_1 and Ca_v_2 voltage-gated Ca^2+^ channels

**Figure 2:**
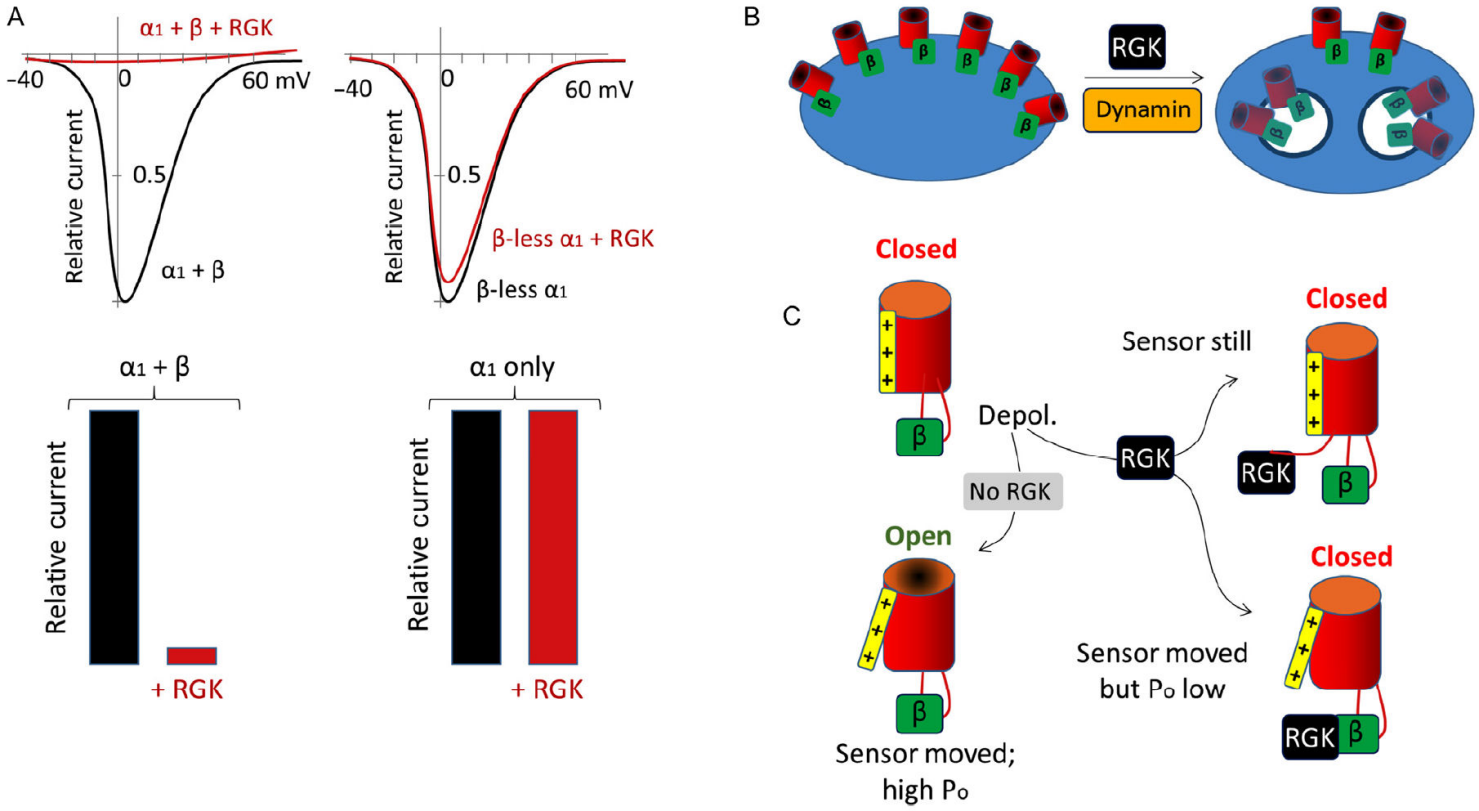
Mechanisms of RGK-mediated inhibition of VGCCs. A, RGKs inhibit VGCC current completely and this inhibition is dependent on the presence of a Ca^2+^ channel β subunit. Thus, β-less channels, which we could generate in macropatches [6], are insensitive to RGK inhibition. B, RGKs exert a dynamin-mediated inhibition of VGCC surface expression. This inhibition depends on RGK-Ca_v_β binding. C, Two modes of RGK inhibition of membrane-resident VGCCs. The left two panels show normal channel opening upon depolarization (Depol.) In the presence of RGK (right two panels), the voltage sensor movement can be blocked, which may not require RGK-Ca_v_β binding, or the voltage sensor may be free but channel P_o_ is decreased. The latter requires RGK-Ca_v_β binding [13].

In a yeast two hybrid screen designed to identify novel Ca_v_β binding partners in β-pancreatic cells, Beguin et al. [18] identified Gem. Its coexpression with L-type channels Ca_v_1.2 and Ca_v_1.3, in the presence of Ca_v_β, led to a dramatic inhibition of currents. The related monomeric GTPase RIN failed to reproduce this inhibition, suggesting that inhibition by Gem was specific. Ever since, many groups have demonstrated direct binding between all RGK proteins and all Ca_v_β subunits, as well as almost complete inhibition of all Ca_v_1 and Ca_v_2 channels, in a variety of expression systems [1,5,14,18-30]. As we now discuss, RGKs can inhibit both channel surface expression and gating, and in many cases, these two mechanisms act in concert (Figure 2).

### 2.1 RGK proteins can inhibit channel surface expression

By analyzing membrane surface expression of extracellularly HA-tagged Ca_v_1.2 channels, Beguin et al. [18,20,31] showed that all RGKs decrease surface expression of L-type channels in PC12 or HEK293 cells.

Other examples exist (Table 1), where, to name two, viral transduction of Rad into guinea pig cardiomyocytes decreases Ca_v_1.2 surface expression [29], and Gem decreases Ca_v_1.2 channel localization at the membrane of tsA201 cells [33]. Other investigators, however, showed that neither Rem nor Rem2 inhibited Ca_v_1.2 channel surface expression in adult guinea pig heart cells or MIN6 cells, respectively. Rather, inhibition of membrane-resident channels took place [25,34]. In addition, Rem2 did not inhibit the surface expression of N-type calcium channels in tsA cells, at a time when calcium currents were dramatically reduced [22].

A reconciliation between these disparate findings was offered by the Colecraft group, which used quantum dots and cell sorting analyses of surface-labeled Ca_v_1.2 channels, to screen thousands of HEK 293 cells [1]. As it turned out, Rem partially reduced surface expression of Ca_v_1.2 to ~40%. Since Ca_v_1.2 currents were completely inhibited, this suggested that both inhibition of surface expression and inhibition of membrane-resident channels took place. Furthermore, the reduction of surface expression was dependent on dynamin, a molecular motor that promotes endocytosis. Thus, in the presence of Rem, a dominant negative dynamin construct was able to restore Ca_v_1.2 surface expression to normal levels [1]. This suggests that RGK proteins likely exert their effect on backward, not forward protein trafficking. In addition, this finding provides a link between RGKs’ two known functions, i.e., regulation of cytoskeletal dynamics and inhibition of VGCCs. This experiment was remarkable for another reason: cells expressing Rem and the dominant negative dynamin still had strongly reduced VGCC currents, suggesting that membrane-resident channels were inhibited via an alternative mechanism. Similar dual mechanisms were also found for Rem2 inhibition of Ca_v_1.2 [2]. Thus, we now know that RGK proteins inhibit VGCCs simultaneously using a slow, trafficking-dependent mechanism and a fast mechanism that inhibits membrane-resident channels (see below and Figure 2).

### 2.2 All RGK proteins can inhibit membrane-resident calcium channels

The Andres and Ikeda groups first showed that RGK proteins can inhibit VGCCs without decreasing channel surface expression. Thus, Rem inhibited L-type currents in β-pancreatic cells and Rem2 inhibited N-type currents in neurons, both without affecting channel surface expression [22,25]. However, the first direct evidence that RGK proteins can inhibit membrane-resident channels came from studies in macropatches. We coexpressed Ca_v_2.1 channels with Ca_v_β_3_ in *Xenopus* oocytes and excised large membrane patches containing these channel complexes. Application of a purified Gem protein to the intracellular face of the macropatches elicited partially reversible channel inhibition, demonstrating unequivocally that membrane-resident channels can be inhibited [6]. In addition, the speed of inhibition was relatively fast, reaching a maximum within 3 min of Gem application. Similarly, Colecraft and colleagues [30] showed that L- and N-type channels could be inhibited minutes within inducing a genetically modified Rem to translocate from the cytoplasm to the membrane.

How are membrane-resident channels inhibited? The Colecraft group demonstrated that Rem employs at least two separate mechanisms for the inhibition of membrane-resident channels: immobilizing the voltage sensor and decreasing channel open probability (P_o_, Table 2) [1].

In the case of reducing voltage sensor movement, Yang et al. [1] used a clever tactic where they compared, on the one hand, the effect of Rem on gating currents (which reflects both the number of channels on the membrane and the mobility of their charged voltage sensors), and on the other hand, the effect of Rem on reducing the number of membrane-resident channels in flow cytometry experiments. This comparison revealed that Rem immobilizes voltage sensor movement of Ca_v_1.2 channels. Thus, in the presence of Rem, as well as the dominant negative dynamin mutant that rescues channel surface expression, gating currents were still reduced. This indicates that voltage sensor movement is obstructed in the presence of Rem and establishes a new mechanism of RGK inhibition of VGCCs. Similar reduction of voltage sensor movement may be exerted by Rad, but not Rem, on native Ca_v_1.1 skeletal muscle channels [35]. However, this has not been differentiated from a possible reduction in the number of available channels on the membrane. Finally, a recent report studying Ca_v_1.2 currents in cardiac myocytes from Rad knockout mice found that Ca_v_1.2 activation is shifted to more negative voltages (channels are easier to open), which is consistent with a retarding effect of Rad on voltage sensor movement. It remains to be determined how universal this effect is [39].

Interestingly, Gem and Rem2 do not seem to inhibit voltage sensor movement in Ca_v_1.2 channels. As will be discussed later, this difference is thought to result from their inability to bind to the channel’s N-terminus [2]. The mode of inhibition in this case likely involves a decrease in surface expression coupled with a decrease in channel P_o_ [1,2]. Indeed, the comparison between Ca_v_1.2 gating currents and tail currents (the latter quantify the total ionic flow through open channels), revealed that channel P_o_ is reduced in the presence of Rem [2]. Similarly, Rem2 was found to inhibit Ca_v_2.2 channels by rendering them nonconducting, the molecular mechanism of which is yet to be studied [22]. It would be interesting to perform single channel recordings to gain deeper insight into the mechanism of P_o_ reduction.

## 3 The role of Ca_v_β in RGK inhibition of VGCCs

RGK inhibition of VGCCs is multifaceted, affecting the surface expression and biophysical properties of membrane-resident channels. But regardless of the mechanism, the presence of Ca_v_β is required for all forms of inhibition. However, RGK-Ca_v_β binding is important for some but not all forms of inhibition, as we discuss below.

### 3.1 RGK-Ca_v_β binding

**Figure 3:**
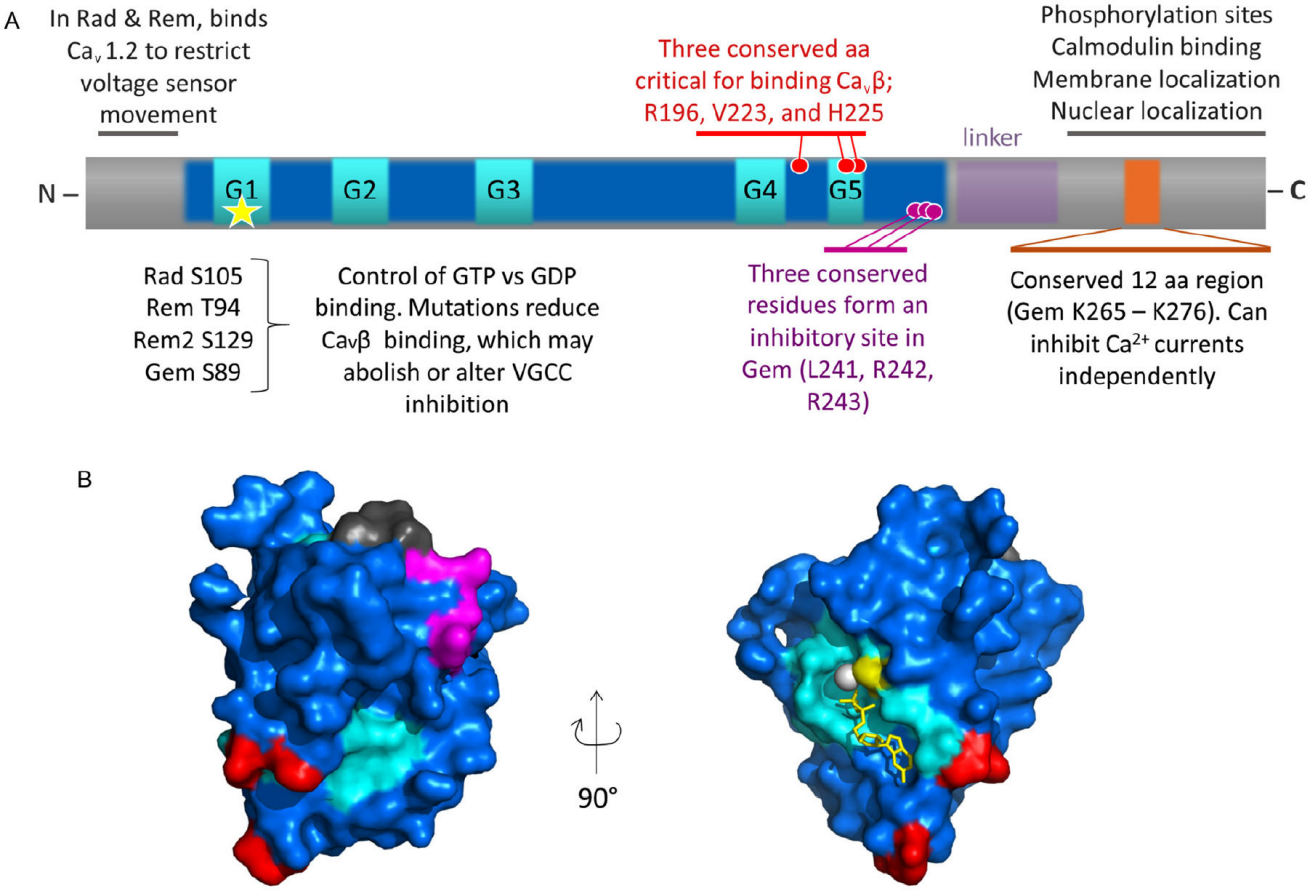
RGK domain organization and structure. A, Schematic diagram denoting the main regions of RGKs. The function of each domain, pertaining to some but not necessarily all RGKs, is indicated. The star indicates the location of a serine critical for GTP/GDP binding. Gray regions, N- and C-termini; blue, RGK core domain; cyan, G1–G5 regions; red and magenta circles, particular residues with the indicated functions; orange, a critical part of the C-terminus. B, The structure of Gem in complex with GDP (yellow sticks, PDB 2HT6). Gray, N-terminus; blue, Gem core; magenta, inhibitory site; red, Ca_v_β binding sites; cyan, G1, G3 and G5 (G2 and G4 are blue); yellow residue is Gem S89; white sphere, Mg^2+^ ion. The C-terminus is not included in the structure.

RGK proteins interact directly with Ca_v_β both *in vitro* and in cells [5,14,18,20,21,23,25-27,30,31,40], and this interaction is promiscuous whereby any RGK protein can interact with any full-length Ca_v_β. This binding was initially proposed to inhibit VGCCs by competing Ca_v_β away from the calcium channel complex and sequestering Ca_v_β into the nucleus [31]. But we now know that this is an unlikely mechanism of inhibition for several reasons. First, when a nuclear export signal is engineered into Rem to prevent it from entering the nucleus and sequestering Ca_v_β with it, it was still able to inhibit VGCCs [1]. Second, a structural model of the Gem-Ca_v_β_3_ interaction has been developed using homology modeling [40] based on Ca_v_β crystal structures [41-43] and a structure of GDP-bound Gem (PDB 2G3Y), as well as on systematic mutagenesis analysis. This model shows that Gem binds to the β_3_ GK domain at a site distinct from the AID-binding pocket, with residues D194, D270 and D272 in β_3_ and R196, V223 and H225 in Gem critical for this interaction (Figure 3, red residues).

Thus, it is unlikely that RGK-Ca_v_β and Ca_v_β-Ca_v_α_1_ bindings are mutually exclusive. Supporting this notion, mutating these critical residues individually or in combination severely weakens or abolishes *in vitro* binding of Gem and β_3_ [6,40], while preserving calcium channel modulation by β_3_. Third, Ca_v_α_1_, Ca_v_β and RGK proteins can form a trimeric complex *in vitro* and in cells [5,6,14,30,40].

### 3.2 Ca_v_β is required for inhibition

Beguin et al. [18] first demonstrated the critical role of Ca_v_² in RGK inhibition: absent Ca_v_β, L-type channels could not be inhibited by RGKs (Figure 2). This turned out to be the case for other VGCCs [18,24,28]. However, in the absence of Ca_v_β, which is required for calcium channel surface expression, VGCC currents are too small to be measured accurately [3,4]. To overcome this problem, and to provide a direct answer to whether Ca_v_β is required for inhibition, we mutated the ABP of Ca_v_β_3_ (M245A and L249A) to achieve two effects: (i) the mutation was mild enough to allow sufficient Ca_v_β_3_-Ca_v_α_1_ binding to promote channel surface expression in *Xenopus* oocytes, in this case Ca_v_2.1; (ii) at the same time, the weakened Ca_v_β_3_-Ca_v_α_1_ binding allowed us to later wash away this mutant Ca_v_β from a macropatch preparation, leaving β-less channels on the plasma membrane [6]. In support of previous findings, β-less channels could not be inhibited by purified Gem perfused onto the intracellular side of the macropatch. However, when WT Ca_v_β_3_ was perfused onto the macropatch first, Gem could now strongly inhibit the channels in a partially reversible manner. Thus, Ca_v_β is absolutely required for Gem inhibition of Ca_v_2.1 channels [6]. Consistent with this requirement, RGK proteins do not inhibit T-type Ca^2+^ channels, which do not associate with Ca_v_β nor require Ca_v_β for their activity [6,22,24].

Is Ca_v_β required for inhibition because it anchors RGKs to the channel? To answer this question, we simultaneously mutated, based on model predictions and previous biochemical studies [6,40], three residues in each Gem and Ca_v_β_3_ to abolish their mutual interaction (creating Gem_mut3 and β3_mut3) [6]. We then tested for Ca_v_2.1 channel inhibition in whole oocytes and in macropatches. Strikingly, Gem_mut3 was able to inhibit Ca_v_2.1 channels expressed with β3_mut3, suggesting that the Gem-Ca_v_β_3_ interaction is not necessary for current inhibition. To reconcile this result with the finding that the presence of Ca_v_β is required for inhibition (as described above), we proposed a “β priming model” where Ca_v_β is required to unmask an inhibitory site on Ca_v_α_1_. This model implies that Gem can bind Ca_v_α_1_ directly. Indeed, we found that Gem coimmunoprecipitated with Ca_v_2.1, even in the absence of Ca_v_β, suggesting direct Gem-Ca_v_2.1 binding. Interestingly, Crump et al. [38] had found that a C-terminally truncated Ca_v_1.2 is relatively resistant to RGK inhibition, hinting that RGK-Ca_v_1.2 interactions may occur. Thus, while Ca_v_β is necessary for some forms of RGK inhibition, Ca_v_β-RGK binding may not be. Subsequent studies have identified both Ca_v_β- and Ca_v_α_1_-binding dependent mechanisms of RGK inhibition [1,2].

### 3.3 RGK inhibition can be Ca_v_β-binding dependent and/or Ca_v_α_1_-binding dependent

To further investigate the effects of RGKs’ multiple interactions with VGCCs, Colecraft and colleagues [2] expressed Ca_v_1.2 with a triple mutant Ca_v_β_2a_ that cannot interact with RGKs. Upon Rem coexpression, currents were still inhibited, albeit to a lesser extent than in the presence of WT Ca_v_β, suggesting that Rem inhibits Ca_v_1.2 channels using both Ca_v_β-binding and Ca_v_α_1_-binding dependent mechanisms. Further experiments revealed that the reduction of surface expression and of channel P_o_ were critically dependent on binding to Ca_v_β. Thus, translocation of a chemically sensitive Rem construct to the plasma membrane dramatically reduced channel P_o_ only in the presence of a WT Ca_v_β, but not the triple mutant Ca_v_β. In contrast, the immobilization of voltage sensors seems to be dependent on Rem-Ca_v_1.2 binding, as it persisted in the presence of the triple mutant Ca_v_β. Rad can also immobilize voltage sensor movement, and both Rad and Rem exert this effect by binding to the Ca_v_1.2 N-terminus [2]. On the other hand, Rem2 and Gem, which show no direct binding to Ca_v_1.2, rely only on Ca_v_β-binding dependent mechanisms of inhibition: reduction in surface expression and channel P_o_ [2]. In complementary experiments carried on Ca_v_2.2 channels, Rem used only Ca_v_β-binding dependent mechanisms to inhibit Ca_v_2.2 [2]. These and the studies discussed earlier highlight the complexity of RGK regulation of VGCCs and demonstrate that the mechanisms of inhibition are RGK, Ca_v_α_1_ and cell type specific.

## 4 Molecular determinants of RGK inhibition

### 4.1 The Ca_v_α_1_ N-terminus

FRET, co-localization analyses and co-IP experiments show that Rem and Rad (but not Rem2 and Gem) bind to the N-terminus of Ca_v_1.2, but not Ca_v_2.2 [2]. Functionally, this allows Rem and Rad to inhibit voltage sensor movement in Ca_v_1.2. Remarkably, overexpression of the Ca_v_1.2 N-terminus can relieve Rem-mediated channel inhibition, albeit only incompletely since the Ca_v_β-binding dependent mechanisms remain in place.

### 4.2 The Ca_v_α_1_ C-terminus

Yang et al. [2] performed extensive FRET, co-localization and co-IP analyses and showed that there is no appreciable binding between the Ca_v_1.2 C-terminus and any of the four (tagged) RGKs. However, Pang et al. [44] suggested that Rem, Rem2 and Rad bind to the C-terminus of Ca_v_1.2 *in vitro*. In addition, they showed that calmodulin overexpression can partially relieve RGK-mediated inhibition, suggesting that RGKs may be competing with calmodulin for the Ca_v_α_1_ C-terminus. The same group also found that Ca_v_1.2 with a truncated C-terminus is relatively resistant to RGK inhibition [38]. While these results await confirmation, they highlight the growing consensus that RGK-mediated inhibition relies on both Ca_v_β- and Ca_v_α_1_-binding mechanisms.

### 4.3 The Ca_v_α_1_ IIS1-IIS3 region

We were able to render Ca_v_2.1 insensitive to RGK inhibition by replacing its IIS1–IIS3 region with that of a T-type channel (Cav3.1) [6]. This finding suggests importance for this region in RGK-mediated inhibition, although the precise mechanism is unclear. We proposed that the IIS1–IIS3 region serves to transmit inhibition to the channel from the RGK protein and through Ca_v_β, which is bound on the nearby I–II loop. Remarkably, T-type channels became RGK sensitive when their I–II loop, together with the IIS1–IIS3 region, were replaced with those of Ca_v_2.1 [6]. This was not the case when only the I–II loop was transplanted, even though Ca_v_β could bind to this chimeric channel and modulate its gating, suggesting that Ca_v_β is not sufficient for conferring RGK sensitivity and that the IIS1-IIS3 region of Ca_v_α_1_ is critical.

### 4.4 The RGK C-terminus

Several groups have demonstrated that truncating the RGK C-terminus abolishes their ability to inhibit VGCCs [1,6,22-24,30,34]. There may be multiple explanations for this. First, the C-terminus itself may be the inhibitory domain of RGK. Leyris et al. [27] showed that the Gem C-terminus can inhibit Ca_v_2.1 channels in *Xenopus* oocytes. In addition, we performed extensive deletion analyses and found a 12 amino acid (aa) C-terminal region of Gem that, when purified and applied to macropatches, can inhibit Ca_v_2.1 [45]. Interestingly, it was the amino acid content, not the sequence of amino acids that was critical. Though this region is conserved in other RGK C-termini, co-expression of Rem and Rem2 C-termini could not inhibit Ca_v_1.2 and Ca_v_2.2 channels [22,23]. Thus, the effect of this 12 aa fragment may be specific for Ca_v_2.1 channels. Interestingly, mutating this 12 aa site in full-length Gem is not sufficient to abolish inhibition, suggesting the existence of one or more additional inhibitory sites. As discussed below, a candidate inhibitory site has been found in the core region of Gem [45].

A more universal function for the RGK C-terminus in channel inhibition lies in the fact that it contains a polybasic motif used for membrane anchorage of RGKs [14,22,46]. Deleting or mutating the RGK C-terminus abolishes their membrane targeting as well as VGCC inhibition [1,6,22-24,30,34]. Thus, the main function of the RGK C-terminus may be to target RGKs to the membrane, where they can, in a higher effective concentration, inhibit VGCCs. In support of this notion, C-terminally truncated Rem and Rem2 could regain their inhibitory function against Ca_v_1.2 and Ca_v_2.2 channels if they were fused to the membrane targeting sequence of an unrelated protein [22,23]. Interestingly, a mutant Rem (L271G) that is not targeted to the membrane is still capable of inhibiting Ca_v_1.2 channels, albeit incompletely [1,31]. Perhaps this is due to Ca_v_β acting as a membrane anchor for Rem.

A complicating factor in determining the precise role of the RGK C-terminus in VGCC inhibition is that it also contains calmodulin and 14-3-3 binding sites, phosphorylation sites and a nuclear localization signal [13]. The roles of those sites are not very clear. For example, we have found that mutating a calmodulin binding site in Gem (W269G) has no effect on Ca_v_2.1-channel inhibition [45], while the same mutation impaired Gem inhibition of native VGCCs in PC12 cells (reviewed by [14,21]).

Finally, a recent study found that the final 11 residues of all RGK proteins are highly conserved across phyla, with a consensus sequence that can serve to differentiate between RGKs and other Ras-related GTPases. The function of this region, termed C-7 because of a ubiquitous cysteine seven residues from the end, has yet to be determined [47]. It is clear, however, that Gem inhibition of Ca_v_2.1 can proceed without it (see deletion constructs from [45]).

### 4.5 The RGK N-terminus

Beqollari et al. [35] recently identified the N-terminus of Rad as a critical molecular determinant of Rad-mediated reduction in voltage sensor movement of native Ca_v_1.1 channels from muscle. Thus, replacing the N-terminus of Rad with that of Rem, which has no effect on Ca_v_1.1 voltage sensors, abolished the inhibition of voltage sensor movement by the mutant Rad. On the other hand, the N-terminus of Rem harbors a protein kinase D1 phosphorylation site that, when phosphorylated, may relieve Rem inhibition of Ca_v_1.2 and contribute to β-adrenergic signaling in the heart [32]. Finally, other studies have shown that the N-termini of Gem and Rem2 do not contribute to Ca_v_2.1 or Ca_v_2.2 inhibition, respectively [22,45].

### 4.6 The RGK core region

We and others have shown that the core region of Gem, without the N- and C-termini, is incapable of inhibiting Ca_v_2.1 channels; it requires at least membrane anchorage [1] or part of the C-terminus for inhibition [45]. But several C-terminal mutants could still inhibit channels, suggesting there was an inhibitory site in the RGK core. We have identified three conserved amino acids (Figure 3, magenta) in the core of RGK proteins (Gem L241, R242, R243), that may form part of an inhibitory site [45]. When mutated in full-length Gem, inhibition is not abolished, but when these three amino acids are mutated together with the C-terminal 12 aa region, all Ca_v_2.1 inhibition is lost. At the same time, Gem binding to Ca_v_β and Gem binding to Ca_v_α_1_ are preserved. Thus, it appears that there are at least two inhibitory sites in Gem, one in the core region and one in the C-terminus, both contributing independently to Gem inhibition of Ca_v_2.1 [45].

### 4.7 The RGK guanine-nucleotide binding domain

RGKs can be GTP- or GDP-bound, and there are differences in the efficacy with which the two forms inhibit VGCCs. Several groups used mutations homologous to a mutation in Ras (Ras^S17N^), which decrease GTP binding, to examine the role of GTP binding in RGK inhibition of VGCCs. Rad^S105N^ and Gem^S89N^ mutants, which were preferentially GDP-bound, and Rem^T94N^ and Rem2^S129N^, display reduced binding to Ca_v_β [20,21,31]. Functionally, Gem^S89N^ (Figure 3, yellow residue) could not inhibit VGCCs in sympathetic neurons [16], suggesting that inhibition may require GTP binding in this system. Rem^T94N^, on the other hand, could still inhibit Ca_v_1.2 channels expressed in HEK293 cells, but without impacting voltage sensor movement [1]. This is in contrast to results obtained in the heart, where Rem^T94N^ could not inhibit Ca_v_1.2 currents, presumably because heart cells can inactivate GDP-bound Rem or prevent it from inhibiting Ca_v_1.2 channels [34]. Similarly mixed results were obtained for Rad^S105N^, which could not inhibit Ca_v_1.2 channels in HEK 293 cells but increased native calcium currents in heart cells [29], suggesting it acted as a dominant negative molecule. Finally, Rem2 inhibition of VGCCs seems to be insensitive to the type of nucleotide bound [22]. Thus, Rem2^S129N^ inhibited sympathetic neuron currents as strongly as WT Rem2. In addition, dialyzing sympathetic neurons that normally express Rem2, with GDPβs, a non-hydrolysable form of GDP, had no effect on current inhibition.

## 5 Physiological significance of RGK-mediated VGCC inhibition

The physiological significance of VGCC inhibition by RGKs has been recently questioned [48]. This is because RGK GTPases have been implicated in many physiological processes that are, hitherto, unrelated to their function to inhibit VGCCs. These include, for example, effects on cell migration, morphogenesis, differentiation and apoptosis—functions that are mostly carried out through RGK actions on Rho kinases, p53, cyclins and other molecules [13,48]. In addition, most studies use overexpression to study RGK-mediated VGCC inhibition. However, we discuss below several reports that clearly illustrate dramatic physiologically relevant effects following manipulations of endogenous RGK levels. Overexpression studies were reviewed elsewhere [13,14,48,49].

### 5.1 Heart

It has been shown that dominant negative suppression of endogenous Rad in the heart increases L-type Ca^2+^ channel currents and action potential duration in cardiac cells and causes longer QT intervals and arrhythmias [29]. Calcium currents of cardiac myocytes from Rad knockout mice are significantly larger and have a negatively shifted activation curve (channels are easier to open) [39]. In addition, these mycocytes are relatively unresponsive to β-adrenergic modulation. Equally compelling studies show that cardiomyocytes from Rem^−/−^ mice have a smaller twitch amplitude, underlined by calcium current densities that are ~15% reduced compared to WT cardiomyocytes and activation that is shifted ~4 mV to more depolarized voltages [37]. Finally, Rem phosphorylation by Protein Kinase D1 can relieve VGCC inhibition in cardiac muscle, in a signaling pathway downstream of β-adrenergic stimulation [32]. These findings demonstrate a critical role for RGK-mediated VGCC inhibition in regulating cardiac function and homeostasis.

### 5.2 Nerve

Using RT-PCR and microarray analyses, Scamps et al. [50] demonstrated specific upregulation of Gem in dorsal root ganglia following neuronal injury. Furthermore, siRNA against endogenous Gem led to a 55% upregulation of P/Q-type currents. The authors reported that Gem expression after injury functioned to specifically inhibit P/Q-type channels, which in turn inhibited neural branching and likely contributed to the homeostatic mechanisms triggered to promote plasticity and neuroregeneration. Interestingly, the mechanism by which Gem specifically targeted P/Q channels rather than the coexisting native N-type channels seemed to involve a simple dosage effect, whereby P/Q channels were comparatively much more sensitive to Gem than N-type channels. This was demonstrated with a dose-response curve in *Xenopus* oocytes, where the levels of Gem expression could be carefully titrated by injecting different amounts of Gem RNA.

Recently, several reports have focused on the role of RGK proteins, in particular Rem2 [51] and Gem [17], in controlling neuronal morphology. In one study, the effects of the Timothy Syndrome (TS) mutation on dendritic arborization were investigated. TS is a cardiovascular and neurological disorder that causes death by the age of three, primarily due to cardiac arrest. In addition, 80% of TS patients also have autism. The disease is caused by a point mutation in Ca_v_1.2 that slows channel inactivation [52]. In a seminal study, Dolmetsch and colleagues [17] showed that neurons generated from TS patients (from their induced pluripotent stem cells) exhibited an activity-dependent reduction in dendritic arborization compared to WT cells (which showed an increase in dendritic arborization upon stimulation). Remarkably, Gem overexpression prevented the reduction in dendritic arborization of TS cells in a manner that required Gem-Ca_v_β binding. Intriguingly, both the reduced dendritic arborization and its reversal by Gem overexpression were observed with TS Ca_v_1.2 channels that also had mutations blocking the channels’ pore. Thus, Gem has to bind to the VGCC complex, but its alteration of dendritic arborization uses a mechanism that is independent of VGCC channel inhibition. The authors proposed that Gem binding or recruitment to the TS channel was impaired, leading to an increased activity of Rho-kinase and a resultant inhibition of dendritic arborization, whereas in WT cells, Gem recruitment and binding to the channel is more efficient, Rho-kinase inhibition is stronger, and a more vibrant dendritic arborization is observed.

While this study and a similar one that studied Rem2 [51] show a major role for RGKs in altering cell morphology independent of VGCC inhibition, it does not exclude a significant role of VGCC inhibition in contributing to the autistic phenotype. We recently found that Gem inhibited TS currents much more weakly than it did WT Ca_v_1.2 currents [53]. Thus, while a role for Ca^2+^ ions may be excluded in the reduced dendritic arborization of TS cells, it cannot be disregarded in contributing to the overall autistic phenotype in TS patients. A recent combination of systems and computational approaches suggested Ca^2+^ as a central factor in the pathophysiology of autism [54].

Finally, a recent study from the Ikeda group suggested that both RGK binding to Ca_v_β as well as RGK inhibition of VGCCs is over 550 million years old. All three residues in both RGKs and Ca_v_βs involved in Ca_v_β-RGK binding are nearly 100% conserved, and fruit flies as well as zebrafish RGK proteins can inhibit calcium channels of rat sympathetic neurons [47]. Thus, the RGK interaction with and inhibition of VGCCs originated prior to the deuterostome-protostome split and is likely to have physiological significance beyond heart, muscle and nerve functions.

## 6 Future directions

As much as the field of RGK regulation of VGCCs has grown, there are many tantalizing unanswered questions. Like chameleons, RGK proteins alter the mode of their inhibition of VGCCs depending on the cellular context and the Ca_v_ channels they are paired with. It remains to be determined which cellular or experimental factors contribute to the observed discrepancies in the modes of RGK inhibition. These factors may include GTPase activating proteins such as nm23 and proteins that interact with VGCC subunits. New RGK binding partners may be identified through yeast two hybrid or other screens. Considering the newly described role of RGK proteins in shaping neuronal morphology [17,51], it would be interesting to identify further links between calcium channels and cytoskeletal reorganization. It would also be interesting to examine whether RGKs interact with synaptic proteins and regulate synaptic transmission, since RGKs have been identified as critical elements for synapse formation [55]. Furthermore, while studies with inducible RGK-mediated inhibition of Ca_v_ channels have shown promising results [30], studies with inducible knockouts are lacking in this field. Such studies will likely uncover yet unknown roles of RGK proteins in both physiological and pathological settings.

## Figures and Tables

**Table 1:** RGK-mediated inhibition of VGCC surface expression

RGK	VGCC	Mechanism	Tissue/Cells	Refs
Rad	L-type (Cav1.2)	Decreases membrane fraction of channels.	Native guinea pig cardio-myocytes	[29]
	Native PC12 VGCC	Prevents VGCC surface expression through interaction with Cavβ. Subcellular distribution of RGK controlled by CaM and 14-3-3 binding. Binds to and sequesters Cavβ to the nucleus. Interaction with Cavβ did not require CaM or 14-3-3 binding.	COS-1 cells for β-subunit interaction, PC12 cells for current, HEK-293T cells	[21]
Rem	L-type (Cav1.2)	Binding to Cavβ to decreases forward trafficking of channels and enhances dynamin-dependent backward trafficking of channels.	HEK 293 cells	[2]
		Controls VGCC membrane trafficking in response to α_1_-adrenergic signaling.	Neonatal rat ventricular myocytes, HEK293T	[32]
		Enhances dynamin-mediated endocytosis, which is Cavβ binding-dependent.	HEK 293 cells	[1]
Rem2		No report of inhibition of surface expression.		
Gem/Kir	L-type (Cav1.2)	Decreases surface expression.	tsA201 cells	[33]
		Decreases surface expression in a calmodulin-dependent manner.	Xenopus oocytes BHK cells and HEK 293 cells	[18]
	L-type (Cav1.3)	Decreases surface expression in a calmodulin-dependent manner.		

**Table 2:** RGK-mediated inhibition of membrane-resident VGCCs

RGK	VGCC	Mechanism	Tissue/cells	Refs
Rad	Ca_v_1.1	Limits voltage sensor movement.	Skeletal muscle	[35]
	Ca_v_1.2, Ca_v_1.3	Reduces channel P_o_ and gating currents.	tsA 201 cells	[36]
Rem	Ca_v_1.1	Lowers probability of channel opening.	Skeletal muscle	[35]
	Ca_v_1.2	Rem knockout increases Ca^2+^ current; has effects on voltage dependence of activation.	Ventricular cardiomyocytes	[37]
		Reduces channel P_o_ (Ca_v_β binding-dependent). Immobilizes voltage sensor (Ca_v_β binding-independent).	HEK 293 cells	[1]
		Lowers channels P_o_.	Adult guinea pig heart cells	[34]
		Decreases Ca^2+^ current when co-expressed with Ca_v_1.2 alone and completely blocks Ca^2+^ current when co-expressed with Ca_v_β.	HIT-T15 cells and embryonic ventricular myocytes	[38]
		Directly binds to Ca_v_β and inhibits ionic current from native channels.	C2C12 myoblasts	[24]
		Decreases P_o_ (Ca_v_β binding-dependent) and immobilizes voltage sensor (Ca_v_β binding-independent).	HEK 293 cells	[2]
	Ca_v_2.2	Inhibits via a Ca_v_β binding-dependent mechanism.	HEK 293 cells	[2]
		Lowers channel P_o_.	HEK 293 cells	[30]
Rem2	Ca_v_1.2, Ca_v_1.3	Completely inhibits Ca^2+^ current without reducing surface expression.	HEK 293 cells	[25]
	Ca_v_2.2	Forms of a non-conducting pore.	Rat DRGs	[22]
Gem/Kir	Ca_v_2.1	Directly inhibits channels in macropatces in a Ca_v_β-dependent but Ca_v_β-binding-independent manner.	*Xenopus* oocytes	[6]
